# Women’s heart disease research in the netherlands: angina with non-obstructive coronary artery disease and beyond

**DOI:** 10.1007/s12471-025-01997-6

**Published:** 2025-10-29

**Authors:** Elisa Dal Canto, N. Charlotte Onland-Moret, Sanne A. E. Peters, Bryn Hummel, Irene G. van Valkengoed, Paula M. C. Mommersteeg, Jeanine Roeters-van Lennep, Marte van der Bijl, Chahinda Ghossein-Doha, Frans H. Rutten, Yolande Appelman, Julie A. E. van Oortmerssen, Maryam Kavousi, Diantha J. M. Schipaanboord, Tijn Jansen, Denise Peeters, Tim R. Sakkers, Elize A. M. de Jong, Behruz Yosofi, Veroni van Es, Irene Göttgens, Tim P. van de Hoef, Sabine Oertelt-Prigione, Eric Boersma, Peter Damman, Hester M. den Ruijter

**Affiliations:** 1 Laboratory of Experimental Cardiology, University Medical Center Utrecht, Utrecht University, Utrecht, The Netherlands; 2 Julius Center for Health Sciences and Primary Care, University Medical Center Utrecht, Utrecht University, Utrecht, The Netherlands; 3 Department of Public and Occupational Health, Amsterdam UMC, Location AMC, Amsterdam, The Netherlands; 4 Center of Research on Psychological Disorders and Somatic Diseases (CoRPS), Department of Medical and Clinical Psychology, Tilburg University, Tilburg, The Netherlands; 5 Department of Internal Medicine, Erasmus MC, University Medical Center, Rotterdam, The Netherlands; 6 Thorax Center, Department of Cardiology, Erasmus Medical Center, Cardiovascular Institute, Rotterdam, The Netherlands; 7 Department of Cardiology, Amsterdam University Medical Center, Location VUmc, Amsterdam, The Netherlands; 8 Department of Epidemiology, Erasmus MC, University Medical Centre Rotterdam, 2040, 3000 CA Rotterdam, The Netherlands; 9 Department of Cardiology, Radboudumc, Nijmegen, The Netherlands; 10 Dutch Heart Foundation (Hartstichting), The Hague, The Netherlands; 11 Gender Research Unit, Department of Primary and Community Care, Radboud University Medical Center, Nijmegen, The Netherlands; 12 Sex- and Gender-Sensitive Medicine Unit, Medical Faculty OWL, Bielefeld University, Bielefeld, Germany

**Keywords:** Women’s Health, Coronary Artery Disease, Sex Characteristics, Coronary Heart Disease, Ischemic Heart Disease, Delivery of Health Care

## Abstract

Cardiovascular disease in women has historically been underrepresented in research. In recent years, several funding bodies, including the Dutch Heart Foundation, have launched numerous research initiatives and consortia in the Netherlands to address knowledge gaps in women. This article provides an overview of the current landscape of cardiovascular disease in women and emphasizes the critical need for continued investment in this field. One area with urgent knowledge gaps is the early detection, diagnosis, therapy, and prognosis of Angina with Non-Obstructive Coronary Arteries (ANOCA) in women with persistent signs and symptoms of ischemia. In the Netherlands, in recent years, we have established a robust clinical infrastructure and a translational framework that enables us to address these challenges. Additionally, we have performed implementation studies to fast-track knowledge on ANOCA in clinical practice, giving us a unique opportunity to transform clinical care for women with signs and symptoms of ischemia. We advocate for a broad perspective that incorporates characteristics such as ethnicity, socio-economic background, and female-specific risk factors. Our goal is to provide solid evidence to ensure the best possible care for all women suffering from persistent signs and symptoms of ischemia.

**Supplementary Information:**

The online version of this article (10.1007/s12471-025-01997-6) contains supplementary material, which is available to authorized users.

## Infobox

**Sex and Gender in Cardiovascular Research**

This article distinguishes between sex and gender to frame our understanding of cardiovascular disease (CVD). *Sex *refers to the biological and physical characteristics that differentiate males from females. These include chromosomes, hormone levels (e.g., oestrogen, testosterone) and reproductive systems. *Gender* refers to the roles, behaviours, identities, and expectations that societies attribute to individuals based on their perceived sex. In this context, gender is influenced by cultural, societal, and personal factors and can vary across different cultures over time. We acknowledge that sex and gender are intertwined and often influence each other in various ways and jointly affect CVD.

## The historical perspective

From the early cohort studies in the 1950s, it became evident that CVD, especially coronary heart disease (CHD), was more prevalent in men than women. As a result, many epidemiological studies did not consider women’s representation or failed to report female data [1]. A typical example of a men-only study is the early Western Collaborative Group Study (1960–1961), which enrolled 3,154 healthy, middle-aged men to study what are now considered ‘classical’ cardiovascular risk factors [2]. Consequently, the impact of CHD in women, the role of female-specific risk factors, and the female-dominant features of CHD in terms of symptom presentation, diagnosis, aetiology, pathophysiology, prognosis, and therapy were largely overlooked.

The exclusion of women extended into early-phase pharmacological trials. Two main concerns drove this: the diethylstilbesterol and thalidomide tragedies in the 1950s–1960s, which caused severe birth defects in children of exposed pregnant women [1]. The second was, concerns that hormonal fluctuations might affect outcomes. Consequently, the 1977 US Food and Drug Administration (FDA) guidelines [3] recommended excluding women with childbearing potential from phase 1 and early phase 2 studies. Women could only be included in later phases once safety and teratogenicity data were provided. In practice, this led to the near-complete exclusion of pre-menopausal women from early clinical trials. Postmenopausal women were also underrepresented, partly due to the lower disease incidence in women and recruitment strategies centred on male disease characteristics.

### The changing landscape: from historical underrepresentation to modern understanding

In the Netherlands, CVD remains the second leading cause of death for both men and women. In 2023, the mortality from CVD was nearly equal in men (19,447) and women (19,303), with the highest rate in women aged 80 + [4]. Over recent decades, the average age at CVD death rose from 73 to 79 in men and 79 to 84 in women. Additionally, women are older at hospital admission and at the diagnosis of acute coronary syndrome (ACS), with a median age of 75 years [4]. Crucially, women experience significantly longer diagnostic delays compared to men (45 versus 33 min) [5].

Recognizing the historical underrepresentation of women in clinical research, the FDA established a committee in 1993 to improve the enrollment of women in clinical trials [6]. This was driven by the concerns over the lack of sex-specific safety and efficacy data and a growing consensus that women should be able to decide whether to participate in early-phase research. Although this marked a shift, progress has been slow. An analysis of cardiovascular drug trials supporting FDA approvals between 2005 and 2015 showed underrepresentation of women in most CVD domains. Adequate participation-to-prevalence ratios (PPR = 0.8–1.2) were observed for atrial fibrillation and hypertension, while women were overrepresented in pulmonary arterial hypertension trials (PPR > 1.2). In contrast, underrepresentation (PPR < 0.8) persisted in heart failure (HF), coronary artery disease (CAD), and ACS [7].

Emerging evidence further underscores important sex-specific differences in medication responses. Women may benefit from lower dosages of cardiovascular medications, particularly in HF treatment [8, 9], but are also more prone to experiencing adverse drug reactions, which remain significantly underreported [10, 11]. For instance, in the LoDoCo2 trial, women were more likely than men to permanently discontinue colchicine due to adverse effects, even among those receiving a placebo [12]. Landmark contributions from the Netherlands have additionally highlighted crucial insights: pre-eclampsia substantially elevates future CAD and HF risk in women [13, 14]; adverse drug reactions from HF medications are disproportionately underreported in women [10], and women with non-obstructive CAD face a significantly higher risk of future CVD ([15]; Tab. 1). These insights underscore the need for sex-sensitive recruitment guidelines and personalised treatment strategies before enrolment even begins.

**Table 1:** Key findings from landmark consortia and projects. Consortia and Initiatives that the Dutch Heart Foundation has supported over the past years on the theme of women’s cardiovascular health.

Consortium/Project (year)	Focus area(s)	Key findings/contributions
Dutch national consortium to promote cardiovascular healthy aging in women: CREW (2013–2018)	Preeclampsia and coronary atherosclerosis	Women post-preeclampsia without ischemic symptoms had more coronary atherosclerosis than women with normal pregnancies [13]
	Premature ovarian insufficiency (POI)	Women with premature ovarian insufficiency had worse CV risk factors but lower cIMT and similar 10-year CVD risk and health score (S18)
	Polycystic Ovary disease (PCOS)	Despite an increased BMI and waist circumference, middle-aged women with PCOS had a moderately unfavorable cardiometabolic profile compared to age-matched controls, a similar 10-year CVD risk score, and lower IMT compared with controls from the general population (S19)
Improving diagnosis of CVD in women: Queen of Hearts (2013–2018)	Heart failure risk post-preeclampsia (PE)	Cardiac remodeling observed in women post-PE, indicating a higher risk for heart failure [28]
	Prediction models	Prediction models for HFpEF [27] and subclinical heart failure and hypertension after pregnancy [26] (S20)
	Cardiovascular risk management	Empirical data to direct guidelines on cardiovascular risk management after PE (S21)
	Adverse drug reactions in women (ADRs); ADR reporting in ACE inhibitors	Identified underreporting of ADRs in HF medications; collaboration with WHO database confirmed higher ADRs in women, especially of reproductive-age [11] (S22)
		Women reported more ADRs with ACE inhibitors compared to men (S23)
Preventing cardiovascular disease in women and their offspring through lifestyle interventions before and during pregnancy: WOMB (2013–2018)	Preconception lifestyle intervention	RCT in obese infertile women showed preconception weight loss improved long-term cardiometabolic health (S24, S25)
	Maternal insulin resistance and neonatal birth weight	Preconception insulin resistance associated with higher neonatal birth weight (S26)
	Cardiac outcomes in offspring	Lifestyle intervention improved cardiac structure/function in children—first human evidence for intergenerational CVD risk reduction (S27)
National collaborative knowledge platforM to imPact on sex- and gendeR sEnSitive cardiovaScular medicine: IMPRESS (2020–2025)	Inflammation and HFpEF	Identified inflammation’s role in cardiac remodeling and HFpEF in women (S28)
	ANOCA and coronary microvascular dysfunction	Created e‑learning for GPs, launched NL-CFT registry, developed non-invasive tools for diagnosis (S29–S32)
	Implementation of Diagnostics	Conducted studies on barriers and facilitators for ANOCA diagnostics implementation (S30–S32)
	Long-term risk in ANOCA patients	Linked angiography data with GP records; found high cardiovascular care use and elevated risk of heart failure among patients with non-obstructive CAD [15]
	Electrocardiographic predictors in ANOCA	ECG patterns (e.g., ischemic signs) were explored for identifying coronary vascular dysfunction in primary care settings (61) (S11)
	Long-term risk in ANOCA patients	Linked angiography data with GP records; found high cardiovascular care use and elevated risk of heart failure among patients with non-obstructive CAD [15] (S7)
	Sex- and ethnic variation in (reasons for) delays in care	Data from the multi-ethnic HELIUS cohort was studied to discover sex- and ethnic differences in delays in care. Qualitative data was collected and studied to discover reasons for delays in care in women and men in a multi-ethnic population, and health-information preferences in this population, which led to the development and implementation of a toolkit to promote symptom recognition and care seeking (S33)
feMAle under-Representation in Executing Cardiovascular research: Understanding Reasons for In-Equality MARIE CURIE (2022–2027)	Underrepresentation in trials	Studied discontinuation rates and barriers in female participants; found women more likely to discontinue colchicine and placebo [12]
Sex differences in tHE Pathophysiology, moleculaR processes and biomarker levels to prEDICT new onset Heart Failure in women (SHE-PREDICTS-HF)	Heart Failure in Women	Link between microvascular endothelial dysfunction and HFpEF (S2)

The table summarizes the major funded consortia, their focus areas, and key findings. Where applicable, multiple findings from a single consortium are presented to illustrate the breadth of impact

CVD cardiovascular disease, CAD coronary artery disease, HFpEF heart failure with preserved ejection fraction, BMI body mass index, GP general practitioner. ACE Angiotensin-converting enzyme, ANOCA angina with non-obstructive coronary arteries, POI Premature ovarian insufficiency, PCOS Polycystic Ovary Syndrome, PE pre-eclampsia, ADRs adverse drug reactions, NL-CFT Netherlands Registry of Invasive Coronary Vasomotor Function Testing, IMT intima-media thickness

### Role of the dutch heart foundation: crucial initiatives in women’s cardiovascular health

**Fig. 1:**
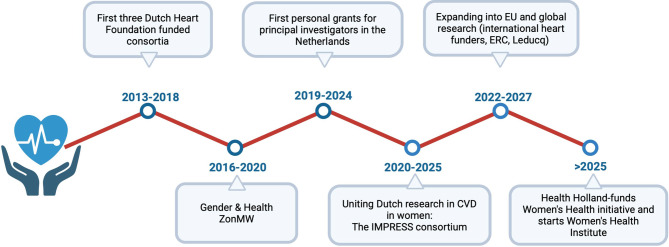
Cardiovascular disease in women: research initiatives in the Netherlands. Overview of the major research programs, consortia, and initiatives funded by the Dutch Heart Foundation since 2013 that have addressed sex- and gender-specific aspects of cardiovascular disease in women

The Dutch Heart Foundation, established in 1964 to reduce the burden of CVD in the Netherlands, has long supported research across prevention, (early) diagnosis, treatment, and patient management (Fig. 1). In 2013, it launched the first dedicated research consortium on women’s heart health, following a research agenda that prioritized CVD in women. Since 2015, the foundation has required that all funded research stratify data by sex.

Shortly after, a national health program from the Netherlands Organization for Health Research and Development (ZonMw) inspired an additional Gender and Health program dedicated to improving, among others, CVD in women. From 2020 onwards, various funders awarded both personal and international grants targeting CVD in women (*Table S1)*. ZonMw also developed online teaching materials to support researchers integrating sex and gender perspectives into research (Infobox; [16]). In 2024 the Dutch Heart Foundation published its new cardiovascular agenda, emphasizing future-proof healthcare, prevention, and the importance of acknowledging individual differences such as sex and gender, socio-economic position, migration background and health understanding. The same year, the Erasmus MC launched the Netherlands Women’s Health Research & Innovation Center, dedicated to improving women’s health outcomes across the life course (Tab. 1).

## Cardiovascular risk management (CVRM) in women

The first clinical recommendations for CVRM in women originated from 1999 [17] and were updated in 2020 [18]. Since then, female-specific and traditional risk factors with a greater impact on women than men have been identified. Key modifiable factors include hypertension, smoking, cholesterol, obesity, and diabetes. While these affect both sexes, the magnitude of the associations is not the same [19]. Large-scale meta-analyses have shown that diabetes confers a 44% greater excess risk of CHD in women [20]. Mechanisms are unclear but may involve biological or care-related factors. Women also achieve better blood pressure control, possibly due to differences in drug type and dosage [21], and show sex-specific vascular responses to smoking [22], potentially explaining their increased risk.

Female-specific factors related to pregnancy, reproduction, and hormonal changes, such as adverse pregnancy outcomes, polycystic ovary syndrome, and menopause timing, have been linked to long-term CVD risk [23, 24]. In 2013, the Dutch Heart Foundation launched new consortia co-led by experts in gynaecology. A key early study showed a linear increase in cardiovascular mortality with earlier age at menopause [25], but many underlying mechanisms remain unclear. Our studies have advanced knowledge on the long-term cardiovascular consequences of hypertensive pregnancy disorders [14, 26, 27], yielding prediction models to guide screening and follow-up after complicated pregnancies [28].

While observational studies have advanced the field, they are inherently limited by residual confounding or reverse causation. Moreover, many are underpowered to detect sex-specific effects. To address this, we performed several Mendelian randomisation studies and found no sex differences in the genetically proxied relationship between diabetes, age at menopause, and CHD [16, 19, 29, 30]. However, body mass index had a stronger causal effect on diabetes risk in women [31]. Similarly, age at menarche and pre-eclampsia were also causally linked to increased CVD risk [32, 33]. Complementing these findings, coronary CT angiography showed atherosclerosis in 30% of women aged 45–55 with prior preeclampsia, versus 18% of controls [13]. Platelet RNA profiles in these women showed enrichment for CAD susceptibility genes [34], contributing evidence to the link between preeclampsia and CAD. While CAD and HF have distinct mechanisms, preeclampsia also correlates with higher HF risk [14, 35], highlighting the need for broad preventive measures for both CAD and HF in this population.

### Clinical presentation of CAD in women

Women differ from men not only in cardiovascular risk profiles but also in symptom presentation. Although chest pain is common to both sexes, women with ACS often report less typical symptoms such as atypical chest discomfort, fatigue, or dyspnoea [36]. In the Netherlands, general practitioners (GPs) are the first point of contact for patients with chest pain. Each year, approximately 700,000 individuals present with such complaints, making up 44% of all chest pain cases in primary care. Yet only 15% are referred to cardiology [37], and ultimately, just 6.6% are diagnosed with CVD [38]. This pathway creates a diagnostic gap, particularly for women with ischemic symptoms but normal or near-normal angiographic findings, many of whom have ANOCA and are misclassified as having non-cardiac chest pain.

To address this issue, the IMPRESS consortium has co-developed an accredited e‑learning program for GPs. This program trains providers to recognize key features of ANOCA—such as prolonged rest-related chest pain accompanied by fatigue—and to distinguish these from other causes like reflux or musculoskeletal complaints. This program, accessible online, aims to reduce misdiagnosis and improve outcomes [39].

### Obstructive CAD

**Fig. 2:**
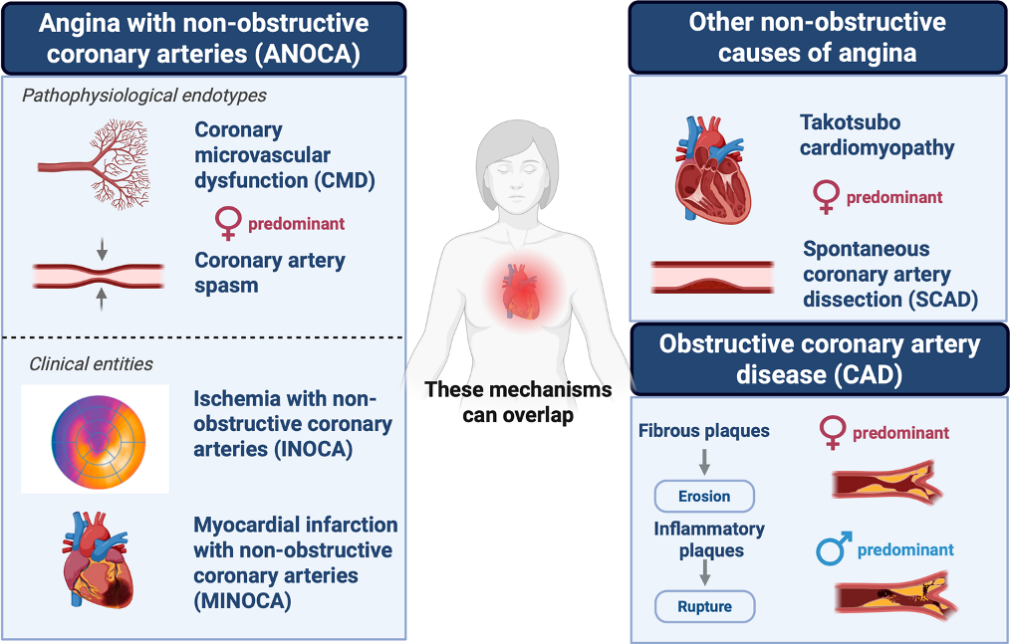
Spectrum of obstructive and non-obstructive coronary artery disease. Schematic representation of the continuum of coronary artery disease (CAD) encompassing both obstructive and non-obstructive phenotypes. The diagram distinguishes obstructive CAD from non-obstructive forms, which include endotypes such as coronary microvascular dysfunction and vasospasm, grouped under the term ANOCA, as well as other entities (Takotsubo cardiomyopathy and spontaneous coronary artery dissection)

Atherosclerotic plaques in women and men differ in composition and behaviour. In the setting of ACS, women often present with plaque erosion rather than plaque rupture compared to men [40], and tend to have fibrous plaques whereas men more frequently present with unstable and inflammatory lesions (Fig. 2; [41]). Also, plaque haemorrhage is associated with plaque vulnerability and adverse outcomes in men, but not in women, suggesting sex-specific differences in the prognostic value of plaque features [42]. As one of the breakthroughs in understanding sex differences in atherosclerosis, the identification of cellular and molecular drivers of atherosclerotic plaques in women in two independent vascular tissue biobanks explains why plaques can differ between sexes [43, 44]. In women, plaque gene expression was enriched for vascular cell plasticity processes like endothelial-to-mesenchymal transition. In men, immune-related pathways predominated. Notably, even in postmenopausal women (~ 2 years), several gene expression patterns were still linked to oestrogen signalling, suggesting long-lasting effects of sex hormones. Sex differences were also evident in vascular gene expression studies using endothelial cells from opposite-sex twins and adults, showing that 14–25% of the endothelial cell gene expression is sex-biased, identifying both innate (likely sex chromosome-based) and acquired (likely sex hormone-based) sex differences. Genes with acquired sex-related differences in adults were indeed more likely to be influenced by sex steroids [45].

Together, these findings indicate that sex differences in vascular biology are present from birth and evolve throughout life, likely contributing to the distinct pathways of CAD in women and men.

### Non-obstructive CAD

Non-obstructive CAD, defined as < 50% luminal stenosis, is more frequently diagnosed in women than in men [46]. In the IMPRESS—Julius GP data-linkage (*n* = 2,546), 39% of women versus 23% of men had non-obstructive CAD. During follow-up, these patients reported persistent symptoms, high healthcare use, as well as an increased HF risk [15]. The term angina with non-obstructive coronary arteries (ANOCA) is a broad, symptom-based umbrella that describes angina in the absence of significant epicardial obstruction. The clinical spectrum of ANOCA includes two groups: ischemia with non-obstructed coronary arteries (INOCA)—characterized by objective evidence of myocardial ischemia such as abnormal stress test, coronary function test (CFT), or perfusion MRI without myocardial injury—and myocardial infarction with non-obstructive coronary arteries (MINOCA) involving acute myocardial injury confirmed by biomarkers or imaging evidence of necrosis. Additionally, spontaneous coronary artery dissection (SCAD) and Takotsubo cardiomyopathy (TTC) represent further non-obstructive entities commonly linked to mechanisms such as coronary microvascular dysfunction (CMD), vasospasm, and myocardial stunning (Fig. 2). These conditions predominantly affect younger or perimenopausal women and present atypically, complicating timely diagnosis and optimal management [47]. Despite the absence of obstructive lesions, all these entities impose significant symptom burdens and adverse outcomes.

To address this unmet clinical need, the Netherlands Society of Cardiology (NVVC) published dedicated guidelines on ANOCA in 2020 [48], advocating a structured diagnostic approach with CFT to differentiate vasospastic from microvascular angina endotypes. However, these recommendations are not yet incorporated into the Dutch General Practitioner guidelines (NHG 2019), which continue to advise against cardiology referrals for atypical chest pain frequently observed in ANOCA.

To facilitate clinical implementation and research, the IMPRESS consortium has supported the establishment of the Netherlands Registry of Invasive Coronary Vasomotor Function Testing (NL-CFT), currently involving data from 20 of the 69 hospitals in the Netherlands [49]. This registry provides a strong infrastructure for ongoing ANOCA research, particularly trials. Initial medical therapy typically precedes invasive testing; however, recent ESC 2024 guidelines strongly recommend CFT (Class I, Level B) for patients experiencing persistent angina despite medical treatment [50]. The diagnostic utility of CFT is high, demonstrating vasomotor dysfunction in approximately 78% of tested patients while maintaining a low complication rate (< 1%) (see Electronic Supplementary Material (ESM) in reference S1). Nonetheless, limited availability and associated costs represent significant barriers, motivating further exploration of alternative diagnostic tools such as ECG-based predictors, Holter monitoring, and peripheral vascular assessments (S1–S3). CFT allows clinicians to differentiate CMD from vasospastic angina, the two main ANOCA endotypes (Fig. 2). Differentiating these endotypes has been shown to improve prognosis and quality of life in ANOCA patients (S4). Furthermore, by recognizing the complexity and diagnostic uncertainty faced by patients, their experience can be improved. Therefore, we developed a patient decision aid that is publicly available online to support shared clinical decision-making for the diagnostic trajectory (S5).

## Ethnic and cultural considerations

CVD risk and healthcare access disparities are not only affected by sex and gender but also by ethnic and sociocultural background. These factors intersect with education, language, and social status to shape care experiences, a focus point within the IMPRESS consortium. Insights from the HELIUS cohort in the Netherlands have revealed persistent disparities in the recognition and treatment of myocardial infarction, particularly among women and individuals from ethnic minority groups, such as those of Surinamese, Ghanaian, Turkish, and Moroccan origin (S6). Ethnic minority women with chest pain are less likely to receive preventive care, often due to language barriers, lower health literacy, and sociocultural factors influencing healthcare-seeking behavior (S7). Despite a higher burden of CVD risk factors, these populations remain underrepresented in clinical trials (S7). Understanding these disparities alongside sex and gender differences is crucial for developing inclusive cardiovascular health strategies.

## Discussion

This review highlights the significant advancements made by researchers in the Netherlands, and most recently, the IMPRESS consortium. Despite this progress, ANOCA remains underdiagnosed and undertreated, disproportionately affecting women and significantly impairing their quality of life. Patients often face long diagnostic delays, misattribution of symptoms to psychosomatic causes, and limited access to specialized care (S6). Women with ANOCA frequently experience multiple hospital visits and referrals before receiving an accurate diagnosis, leading to frustration, emotional distress, and decreased trust in healthcare providers (S8). Cardiologists also report challenges in implementing treatment recommendations due to skepticism, variability in diagnostic workups, and the influence of gendered stereotypes in clinical decision-making. The lack of recognition of sex-specific manifestations of CHD, including ANOCA, has likely contributed to its underprioritization in cardiovascular research (S9). Overcoming these barriers requires clinician education, more research funding, and patient-centred strategies. Patients themselves have emphasized the need for clearer diagnostic protocols, improved physician awareness, and more consistent care (S10).

The optimal structure for delivering women-specific cardiovascular care is under debate. Specialized outpatient clinics may improve the recognition and management of conditions such as ANOCA, MINOCA, SCAD, and TTC in women, which often overlap in presentation and are challenging to diagnose. However, specialized clinics may risk underdiagnosis in men with these conditions, underscoring the need for inclusive care models and greater awareness of sex-related pathophysiology.

The role of CFT in clinical practice also remains controversial. While CFT effectively distinguishes between ANOCA subtypes and guides targeted therapies, routine adoption remains limited by accessibility and cost barriers. Although CFT-stratified treatment may relieve symptoms and improve quality of life (S4), evidence regarding its long-term impact on prognosis and major adverse cardiovascular events is still lacking. Recent trials, such as EDIT-CMD, in which commonly prescribed medications like diltiazem failed to improve CMD (S11), and the recently presented WARRIOR trial, which showed no significant difference in major cardiovascular outcomes between intensive medical therapy and usual care in women with suspected INOCA, further underscore the complexity of managing this condition (S12). Additionally, patient pathways are often long and uncertain, underscoring the need for clearer diagnostic protocols and shared decision-making tools to facilitate timely and effective patient-centred care.

Women remain underrepresented in cardiovascular trials for multiple reasons: older age at diagnosis (S13), concerns about adverse effects (S14), caregiving responsibilities (S15), and potentially biased recruitment strategies and physician-patient communication. The MARIE CURIE project currently investigates these factors comprehensively (Tab. 1). Addressing these barriers through targeted recruitment strategies and better patient-provider communication is essential for improving women’s representation in cardiovascular research.

Diagnostic delays in women warrant further study. Such delays partly arise from distinct pathophysiological presentations in women, who frequently exhibit non-obstructive coronary mechanisms undetectable by coronary angiography. Atypical symptom profiles and lack of obstructive lesions lead to misinterpretation or underrecognition, particularly when healthcare providers are inadequately aware of sex differences (S16, S17). Consequently, educational initiatives targeting both clinicians and the public, along with culturally and gender-tailored community-based strategies, remain crucial. Finally, adopting a holistic and inclusive approach, integrating stress, personality, familial predisposition, and socio-economic context, can improve diagnostic accuracy, facilitate timely intervention, and promote equitable outcomes across diverse populations.

### Future research direction

The Netherlands has made early investments in infrastructure for women’s cardiovascular research and recognition of sex-specific disease manifestations. This has been made possible through sustained support from funders such as the Dutch Heart Foundation. Moving forward, key priorities for inclusive cardiovascular care include:

1. Training clinicians and educating the public to improve recognition of the early signs of ANOCA.
2. Advancing non-invasive diagnostic tools such as ECG- and echo-based tools; studying ANOCA outcomes in both sexes; investigating genetic predispositions and pathophysiological drivers; designing patient-centred interventions addressing both medical and psychosocial needs.
3. Strengthening primary care pathways, improving referral strategies that account for intersectionality and atypical presentations
4. Strengthening national networks to promote research funding that explicitly supports diversity-, sex- and gender-sensitive cardiovascular science.
5. Supporting community-based initiatives tailored to cultural needs, for more inclusive and effective care (S9).

## Conclusions

Women with ANOCA have long been underrecognized due to a combination of atypical symptom presentation, limited clinician awareness, and the historical reliance on diagnostic tools optimized for detecting obstructive CAD. As awareness improves, enhanced diagnostic strategies—including CFT and emerging non-invasive approaches—now facilitate the detection of myocardial ischemia in the absence of obstructive lesions. CMD is a key priority due to its burden, diagnostic dilemmas, and poor prognosis. Moving forward, the Netherlands’ robust clinical and research infrastructure should be implemented to refine diagnostic pathways, validate non-invasive tools, and deepen our understanding of sex-specific pathophysiological processes. This should ultimately lead to inclusive, personalized cardiovascular care.

## Supplementary Information

The Supplementary Information includes Supplementary Table 1, listing national and international grants and consortia targeting cardiovascular disease in women in the Netherlands over the past years, together with Supplementary References S1–S33 supporting studies cited in the text.
