# Supplementary material for: Women’s heart disease research in the netherlands: angina with non-obstructive coronary artery disease and beyond
Source: Neth Heart J. 2025 Oct 29;33(12):395–403. doi: 10.1007/s12471-025-01997-6 (PMC12638557; doi:10.1007/s12471-025-01997-6)
Supplement: Supplementary file 1 — The Supplementary Information includes Supplementary Table 1, listing national and international grants and consortia targeting cardiovascular disease in women in the Netherlands over the past years, together with Supplementary References S1–S33 supporting studies cited in the text. [file 12471_2025_1997_MOESM1_ESM.docx]

**Women’s Heart Disease Research in the Netherlands: Angina with non-obstructive coronary artery disease and beyond**

Elisa Dal Canto^1^, Charlotte Onland-Moret^2^, Sanne Peters^2^, Bryn Hummel^3^, Irene van Valkengoed^3^, Paula Mommersteeg^4^, Jeanine Roeters-van Lennep^5^, Marte van der Bijl^5^, Chahinda Ghossein-Doha^6^, Frans Rutten^2^, Yolande Appelman^7^, Julie A.E. Van Oortmerssen^8^, Maryam Kavousi^9^, Diantha Schipaanboord^1^, Tijn Jansen^10^, Denise Peeters^10^, Tim Sakkers^1^, Elize de Jong^1^, Behruz Yosofi^10^, Veroni van Es^11^, Irene Göttgens^12^, Tim van de Hoef^1^, Sabine Oertelt-Prigione^12,13^, Eric Boersma^6^, Peter Damman^10^*& Hester den Ruijter^1^*

*Both authors contributed equally

**Supplementary Material**

**Supplementary sTable1.** Personal and international grants targeting cardiovascular disease in women awarded in the Netherlands over the past years.

| **Title of the project** | **Year** | **Institute** |
| --- | --- | --- |
| ***Knowledge syntheses of DHF in collaboration with the Gender and Health program of the Netherlands Organisation for Health Research and Development* (ZonMw)** | | |
| From gender-sensitive psychosocial factors to diversity in incidence and outcomes of ischemic heart disease: a systematic review and meta-analysis | 2017-2018 | Tilburg University |
| Knowledge synthesis for coronary microvascular disease: a gender-specific framework | 2017-2019 | ErasmusMC |
| PReclinical dIastolic Dysfunction and its progression to heart failure with preserved ejection fraction in women and mEn (PRIDE) | 2017-2019 | UMC Utrecht |
| Sex-specific net clinical effect of the “Big Five ”cardiovascular drugs: systematic review and quantitative meta-analysis | 2017-2018 | ErasmusMC |
| SUStainable lifestyle inTERventions in (peri)menopausal women at high risk for, or newly diagnosed with, CVD. What’s the evidence? (SUSTER) | 2017-2018 | NIVEL |
| ***Call 2019 ‘Gender en Health Implementation’ in collaboration of DHF with the Gender and Health program of the Netherlands Organisation for Health Research and Development* (ZonMw)** | | |
| VOORWAARTZ - het in kaart brengen van behoefte en passendheid als VOORWaarden voor implementatie van een sekse-en gendersensitief Algoritme voor het uitsluiten van coronAiRe vaaTZiekten | 2019-2020 | UMC Utrecht |
| Hart voor vrouwen in Den Haag | 2019-2020 | LUMC |
| Cardiovasculair risicomanagement in de eerste lijn voor vrouwen | 2019-2020 | UMC Utrecht |
| Leefstijladvies op maat voor moeders met een gecompliceerde zwangerschap | 2019-2020 | Erasmus MC |
| Gender differences in patients with an acute coronary syndrome in The Netherlands: a retrospective study. | 2015-2016 | Amsterdam UMC |
| Cardiac symptoms and Menstruation: A risky period? | 2018-2019 | ErasmusMC |
| PlacEntal Acute atherosis RefLecting Subclincal atherosclerosis (PEARLS) | 2018-2020 | Maastricht University |
| Hacking Women's Stroke - Big data for precision stroke prevention | 2018-2020 | LUMC |
| Alles heeft een ritme: preventie van atriumfibrilleren encomplicaties in vrouwen en mannen | 2019 - 2020 | Erasmus MC |
| Sekse- & genderspecifieke gezondheidsdeterminanten en de impact op de transitie van ziekte naar multimorbiditeit (SHIFT) | 2019 - 2020 | Erasmus MC |
| Implementatie leidraad microvasculair coronairlijden in decardiologische praktijk | 2019 - 2020 | Erasmus MC |
| **The personal grants of the Dutch Heart Foundation (Dekker)** | | |
| The influence of sex steroids on blood platelets (trans-study) | 2018-2020 | AmsterdamUMC |
| Hacking Women's Stroke - Big data for precision stroke prevention | 2018-2020 | LUMC |
| Pulmonary artERial hypertenSion: bonE morPHOgeNetic protein and Estrogen signaling out of control (PERSEPHONE) | 2018-2022 | AmsterdamUMC |
| Are men bigger sweet-hearts? Molecular metabolic differences at the basis of gender-specific cardiac pressure overload disorders | 2020-2024 | Maastricht University |
| Towards personalized medicine in chronic heart failure: focusing on sex differences and atrial fibrillation | 2020-2022 | UMCG |
| Prevention of myocardial infarction and primary atherogenic cardiovascular disease from a sex-specific life course perspective: a big-data project | 2020-2021 | LUMC |
| Heart failure and atrial fibrillation: Distinct but not too different? Similar butnot the same? | 2022-2026 | Erasmus MC |
| SAPPHIRE: Spiral Artery Placental PatHology Reflects vascular hEalth | 2025-2028 | Erasmus MC |
| Unravelling the pathogenesis of peripartum cardiomyopathy | 2022-2025 | Maastricht University |
| Sex-Med; sex-specific evidence on heart failure medication safety and effectiveness to support sex-specific treatment recommendations and improve treatment in women | 2024-2027 | Utrecht University |
| FEMININE: explaining the FEMale preponderance of INtracranIal aNEurysms | 2023-2028 | UMC Utrecht |
| Connecting to the core of the matter. Optimizing the diagnosis of myocardial ischemia in angina with no obstructive coronary artery disease. | 2024-2028 | UMC Utrecht |
| **Other personal grants** |  |  |
| Sex Differences in the Risk Factor Profile for Atherosclerosis in Various Vessels | 2016 - 2019 | Veni (ZonMw). Erasmus MC |
| **International consortia (title, year and funding scheme)** | | |
| Atherosclerotic plaque erosion leading to thrombus formation. The role of extracellular vesicles in ENDotheliaL transdiffErentiation, apoptoSiS and leucocyte and platelet adhesion (ENDLESS) | 2018-2021 | European Research Area Network on Cardiovascular Diseases |
| Bi-national investigation of placental pathology and maternal cardiovascular health (BI-PATH) | 2023-2028 | DHF, BHF and DZHK grant |
| Identifying molecular drivers to understand and treat spontaneous coronary artery dissection (ID-SCAD) | 2025-2030 | DHF, BHF, LDF and DZHK grant |
| ROMA WOMEN: Randomized Comparison of the Clinical Outcome of Single vs Multiple Arterial Grafts in Women | 2024-2029 | DHF clinical trial program |

**Supplementary References**

S1. Schipaanboord DJM, Woudstra J, Appelman Y, et al. The Diagnostic Value of ECG Characteristics for Vasospastic and Microvascular Angina: A Systematic Review. Ann Noninvasive Electrocardiol. 2024;29(5):e70003.

S2. Canto ED, van Deursen L, Hoek AG, et al. Microvascular endothelial dysfunction in skin is associated with higher risk of heart failure with preserved ejection fraction in women with type 2 diabetes: the Hoorn Diabetes Care System Cohort. Cardiovascular Diabetology. 2023;22(1):234.

S3. Woudstra J, Vink CEM, Schipaanboord DJM, et al. Meta-analysis and systematic review of coronary vasospasm in ANOCA patients: Prevalence, clinical features and prognosis. Front Cardiovasc Med. 2023;10:1129159.

S4. Ford TJ, Stanley B, Good R, et al. Stratified Medical Therapy Using Invasive Coronary Function Testing in Angina: The CorMicA Trial. J Am Coll Cardiol. 2018;72(23 Pt A):2841-55.

S5. Hartstichting. Problemen in de kleine vaatjes van het hart: coronaire vaatdysfunctie [

S6. Van Schalkwijk DL, Widdershoven J, Elias-Smale S, et al. ShareHeart: A patient journey map of patients with ischemia and non-obstructive coronary artery disease based on qualitative research. J Clin Nurs. 2023;32(13-14):3434-44.

57. Hummel B, Harskamp RE, Vester A, Galenkamp H, Mommersteeg PMC, van Valkengoed IGM. Chest pain in a multi-ethnic population: A community-based study on sex differences in chest pain prevalence and care contacts. Int J Cardiol Cardiovasc Risk Prev. 2025;24:200361.

S8. van Schalkwijk DL, Widdershoven J, Magro M, et al. Clinical and psychological characteristics of patients with ischemia and non-obstructive coronary arteries (INOCA) and obstructive coronary artery disease. Am Heart J Plus. 2023;27:100282.

S9. Modderkolk L, Göttgens, I., van den Hurk, L. & Oertelt-Prigione. INOCA recommendation uptake by cardiologists in The Netherlands: Perceived barriers and facilitators informed by the COM-B Model and Theoretical Domains Framework. . Preprint. 2024.

S10. Bairey Merz CN, Pepine CJ, Walsh MN, Fleg JL. Ischemia and No Obstructive Coronary Artery Disease (INOCA): Developing Evidence-Based Therapies and Research Agenda for the Next Decade. Circulation. 2017;135(11):1075-92.

S11. Jansen TPJ, Konst RE, de Vos A, et al. Efficacy of Diltiazem to Improve Coronary Vasomotor Dysfunction in ANOCA: The EDIT-CMD Randomized Clinical Trial. JACC Cardiovasc Imaging. 2022;15(8):1473-84.

S12. Handberg E PC, editor WARRIOR: Intensive Medical Therapy vs. Usual Care in Women With Suspected INOCA. . American College of Cardiology Scientific Session (ACC.25). 2025 March 29, 2025.; Chicago, IL.

S13. Granbom M, Clemson L, Roberts L, et al. Preventing falls among older fallers: study protocol for a two-phase pilot study of the multicomponent LIVE LiFE program. Trials. 2019;20(1):2.

S14. Harrison JM, Jung M, Lennie TA, et al. Refusal to participate in heart failure studies: do age and gender matter? J Clin Nurs. 2016;25(7-8):983-91.

S15. Spiering AE, van Ommen A, Roeters van Lennep JE, et al. Underrepresentation of women in cardiovascular disease clinical Trials-What's in a Name? Int J Cardiol Heart Vasc. 2024;55:101547.

S16. de Torbal A, Boersma E, Kors JA, et al. Incidence of recognized and unrecognized myocardial infarction in men and women aged 55 and older: the Rotterdam Study. European Heart Journal. 2006;27(6):729-36.

S17. van der Ende MY, Juarez-Orozco LE, Waardenburg I, et al. Sex-Based Differences in Unrecognized Myocardial Infarction. J Am Heart Assoc. 2020;9(13):e015519.

S18. Gunning MN, Meun C, van Rijn BB, et al. The cardiovascular risk profile of middle age women previously diagnosed with premature ovarian insufficiency: A case-control study. PLoS One. 2020;15(3):e0229576.

S19. Meun C, Gunning MN, Louwers YV, et al. The cardiovascular risk profile of middle-aged women with polycystic ovary syndrome. Clin Endocrinol (Oxf). 2020;92(2):150-8.

S20. Mohseni-Alsalhi Z, E BNJJ, Delmarque J, van Kuijk SMJ, Spaanderman MEA, Ghossein-Doha C. Prediction Model Based on Easily Available Markers for Aberrant Cardiac Remodeling in Women After Pregnancy. Hypertension. 2023;80(8):1707-15.

S21. Pre-eclampsia is associated with earlier onset and higher incidence of cardiovascular risk factors [press release]. 4 April 2025 2025.

S22. Bots SH, Groepenhoff F, Eikendal ALM, et al. Adverse Drug Reactions to Guideline-Recommended Heart Failure Drugs in Women: A Systematic Review of the Literature. JACC Heart Fail. 2019;7(3):258-66.

S23. Bots SH, Schreuder MM, Roeters van Lennep JE, et al. Sex Differences in Reported Adverse Drug Reactions to Angiotensin-Converting Enzyme Inhibitors. JAMA Network Open. 2022;5(4):e228224-e.

S24. Wekker V, Huvinen E, van Dammen L, et al. Long-term effects of a preconception lifestyle intervention on cardiometabolic health of overweight and obese women. Eur J Public Health. 2019;29(2):308-14.

S25. van Dammen L, Wekker V, van Oers AM, et al. Effect of a lifestyle intervention in obese infertile women on cardiometabolic health and quality of life: A randomized controlled trial. PLoS One. 2018;13(1):e0190662.

S26. Wang Z, Nagy RA, Groen H, et al. Preconception insulin resistance and neonatal birth weight in women with obesity: role of bile acids. Reprod Biomed Online. 2021;43(5):931-9.

S27. den Harink T, Blom NA, Gemke R, et al. Preconception lifestyle intervention in women with obesity and echocardiographic indices of cardiovascular health in their children. Int J Obes (Lond). 2022;46(7):1262-70.

S28. van Ommen AM, Diez Benavente E, Onland-Moret NC, et al. Plasma Proteomic Patterns Show Sex Differences in Early Concentric Left Ventricular Remodeling. Circ Heart Fail. 2023;16(7):e010255.

S29. Crooijmans C, Jansen TPJ, Konst RE, et al. Design and rationale of the NetherLands registry of invasive Coronary vasomotor Function Testing (NL-CFT). Int J Cardiol. 2023;379:1-8.

S30. Schipaanboord DJM, Jansen TPJ, Crooijmans C, et al. ANOCA patients with and without coronary vasomotor dysfunction present with limited electrocardiographic remodeling. Int J Cardiol Heart Vasc. 2024;50:101347.

S31. Schipaanboord DJM, Jansen TPJ, Scherpenhuijzen L, et al. Ischemia during exercise stress testing, an indication of coronary vasomotor dysfunction? Int J Cardiol Heart Vasc. 2025;56:101580.

S32. Woudstra J, Mourmans SGJ, Vink CEM, et al. Relationship between peripheral and intracoronary blood flow in patients with angina and nonobstructive coronary arteries. Am J Physiol Heart Circ Physiol. 2024;327(4):H1086-h97.

S33. Hummel B, van Oortmerssen JAE, Borst CM, et al. Sex and ethnic differences in unrecognized myocardial infarctions: Observations on recognition and preventive therapies from the multiethnic population-based HELIUS cohort. Int J Cardiol Cardiovasc Risk Prev. 2024;20:200237.
